# The Hospital. Nursing Section

**Published:** 1902-01-04

**Authors:** 


### The Hospital.

### Nursing Section.

Contributions for this Section of "The Hospital" should be addressed to the Editor, "The Hospital" Nursing Section, 28 & 29 Southampton Street, Strand, London, W.C.

No. 797.—Vol. XXXI. *SATURDAY, JANUARY 4, 1902.*

### Notes on News from the Nursing World.

### THE COMING CORONATION.

Early in October a correspondent suggested that it might be possible to make some special arrangement for nurses to see the Coronation procession. In publishing the suggestion, which was accompanied by an appeal to us, we invited the views of our readers, but hinted that it might not be possible to carry them into effect. The letters which we subsequently received showed, as we anticipated, a strong desire on the part of nurses to be provided with seats along the route in such a manner as to form a representative gathering. But, unfortunately, the result of inquiries compels us reluctantly to state that it does not seem practicable to obtain a site that would place it within our power to offer accommodation in a desirable position at a price that would be at all within the limits of a nurse's purse. We make this announcement thus early in order that none of our correspondents should lose any opportunity that may occur of securing individually, or by combination of a party, the much coveted privilege.

### ROYALTY AT CORK.

The Duchess of Connaught paid a visit last month to the Victoria Hospital for Women and Children at Cork. She arrived at noon, accompanied by the Hon. Lady M'Calmont and the Earl and Countess of Bandon, and was received by the ladies' committee, several of whom were presented to her. The Duchess was then conducted through the wards, operation room, etc., by the medical staff and lady superintendent, and expressed herself greatly pleased with all that she saw. At the door of the children's ward her Royal Highness was presented with a beautiful bouquet by Miss Dorothy Cummins on behalf of the children, which the Duchess graciously accepted.

### MARLBOROUGH HOUSE AND THE PENSION FUND NURSES.

The souvenir is gaining in popularity as its excellence becomes known. One who was present on the last occasion writes :—"Your very pleasing Christmas card, the Souvenir, reached me to-day. Thanks so much for it. I think the photos the best copies I have ever seen, and having been a photographer since 1885 I am rather severe on reproductions. The large plate with portraits of the King and Queen is charming ; the likeness of the Founder is excellent."

### PENSION FUND NURSES AND "THE HOSPITAL."

We shall be glad if, in future, all members of the Royal National Pension Fund for Nurses who subscribe to The Hospital will apply for the paper to the Secretary at the offices of the Pension Fund, 28 Finsbury Pavement, and not to the offices of The Hospital, as some of them are in the habit of doing.

### THE WAR NURSES.

The *Avoca* arrived at Southampton from South Africa on December 19th with the following nursing sisters on board :—B. E. Brooks, who rejoins the ship ; R. L. Shappere, who also rejoins the ship ; J. H. M. O'Ryan, who requires two weeks' leave, but will rejoin the ship if leave is not granted ; L. Hand, who requires one month's leave, but will rejoin the ship if leave is not granted ; E. L. Courser, who requires three months' leave on medical certificate ; and T. E. Nixon, who is time-expired, but wishes to rejoin the *Avoca*. All the above are members of the A.N.S.R. The *Nubia* arrived on the 20th ult. with the following as "ship's staff" :—D. F. Palmer. A.N.S. ; J. E. Phillips, A.N.S.R ; M. E. Richards, A.N.S.R. ; L. M. Green, A.N.S.R. ; J. T. Wells, civil nurse ; and J. H. Anderson, civil nurse. All rejoin the ship. The *Tagus* came in on the 23rd ult. with M. Haviland and F. Harvey, both of whom require 30 days' leave before returning to South Africa ; B. L. Bracey, who requires six months' leave, and returns to South Africa ; and K. Driver, who requires six months' leave, and does not wish to return under twelve months. The *Dilwara* arrived on Christmas Eve. The following nursing sisters disembarked :—M. J. Richards, A.N.S., requires two months' leave ; M. I. Burdett, A.N.S.R., requires one month's leave, and does not wish to return to South Africa ; J. Lovett, A.N.S.R., requires one month's leave, and returns to South Africa ; L. Warriner, A.N.S.R., requires one month's leave, and returns to South Africa ; and M. S. Bidmead, South Australian N.S., requires one months' leave, and does not wish to return to South Africa. The *Britannic*, arriving on December 26th, brought two members of the A.N.S.R., viz., A. S. Liell and N. Sutcliff, both wishing to return after one month's leave ; and E. Waller, civil nurse, for duty on voyage, and returning to South Africa.

### DEATH OF THE MATRON OF THE WELSH HOSPITAL.

It is a sad and remarkable fact that every official of the Welsh Military Hospital at Pretoria has failed to survive the war. Miss Marion Lloyd, whose death from enteric fever, we regret to announce, took place last month, went out to South Africa with Dr. Tom Jones, Professor Alfred Hughes, and Sister Sage, all of whom passed away before her. Miss Lloyd was trained at the General Hospital, Bristol. She was afterwards matron of the Cancer Hospital, Manchester, and gave up the important post of matron of Bolton Infirmary to undertake the duties of matron of the Welsh Hospital. Recently she was asked to become matron of the hospital in Bechuanaland, but she elected to remain at Pretoria with the forces until the end of the war. She was awarded the order of the Red Cross for her services, which were of a most valuable character, and it is not too much to say that her decease at the early age of 35 is a severe loss to the profession which she adorned. The eldest sister of Miss Lloyd is Mrs. Mathias, who was for ten years lady superintendent of the Royal United Hospital, Bath, and is now lady superintendent, under the London County Council, at Farmfield, Hookwood, Horley.

### A McKINLEY ORDER OF NURSES.

The popularity of Mr. McKinley in the American nursing world is evinced in a striking manner by a petition which has been presented by the nurses of Boston, Mass., to Governor Crane, asking that an Order of Nurses might be established as a memorial to the late President. It is proposed that the Order should provide skilled nursing for the sick poor on the lines of the Queen Victoria Jubilee Institute in this country.

### DUNDEE AND SOUTH AFRICA.

In addition to the sisters and nurses already mentioned in our columns as having been appointed matrons in the Concentration Camps in South Africa, we understand that Miss Grace M. M. Mackenzie, a former sister of the Royal Infirmary, Dundee, has received a similar appointment, while five other nurses have been accepted for ordinary service in the camps. The matrons and nurses were selected by Miss J. M. Duff, matron of the Royal Infirmary, at the request of Miss E. S. Haldane, Cloanden, Auchterarder (a Manager of the Edinburgh Royal Infirmary) acting on behalf of the Colonial Office.

### THE SCOTTISH BRANCH OF THE JUBILEE INSTITUTE.

At the annual meeting of the Scottish Branch of Queen Victoria's Jubilee Institute, after speeches in praise of the work had been delivered, the report was adopted with enthusiasm. The Scottish Council are at present responsible for 51 nurses and probationers who are employed in different parts of the country. During the year 27 nurses were engaged from the home at Edinburgh by local committees, and 17 entered it for the required month of probation before receiving hospital training. Thirty-one probationers who had received hospital training entered the home for six months' district training, and 28 nurses completed their full training as Queen's nurses at Edinburgh. Four nurses received three months' training in the Edinburgh City Hospital, and 1 received three months' training in the Glasgow Maternity Hospital. Twenty-two nurses resigned—4 for hospital appointments, 8 for private nursing, 1 for colonial nursing, 5 to be married, and 4 to go home. Thirteen new branches engaged Queen's nurses, and 1 affiliated to the Institute. There are now 224 Queen's nurses in Scotland, working under 144 affiliated branches, of which 22 are under two county associations. There is a significant note at the close of the report to the effect that the Scottish Council find that many of the Queen's nurses are tempted to leave the service of the Institute in order to obtain more highly-paid appointments for which their good training and high character make them eligible. Accordingly, they have considered it to the advantage of the work to add the annual interest of £1,000 to the interest of £5,000 set aside by the Council of the Institute for annual gratuities of £5 each to the Queen's nurses in the United Kingdom—according to their seniority on the roll of Queen's nurses.

### PROGRESS IN THE STATES.

An American nurse, who has been describing her experiences of nursing in Porto Rico during the Spanish-American war, the Philippines, and Tien Tsin, found little or no fault with her surroundings or her work. But she was very indignant with the authors of a report that there had been improper conduct on the part of the nurses in the Philippines, and she affirmed that it had its origin " in the actions of the Red Cross representative and those women whom he had with him." The American Red Cross Society will doubtless take note of Miss Chanler's remarks. A more satisfactory statement by her was that she found that many of the physicians who had strongly objected to trained nurses during the Spanish-American war had been completely converted when less than two years later she met them in the Philippines. Miss Chanler was much astonished at the very large number of nurses from the Western States whom she met among army nurses. "Most of them were from training schools of which she had never heard, situated in the Far West; yet she found them, almost without exception, excellently instructed and doing the most satisfactory work."

### THE VICTIMS OF THE RAILWAY ACCIDENT AT LIVERPOOL.

Following the report of a bad accident on the Overhead Railway in the tunnel by the Dingle Station, the ambulance from the Royal Southern Hospital was sent for at once, the authorities expecting several victims, but only four were brought in. Two men and a boy were suffering mostly from the effects of the foul air and almost intolerable suffocation. Naturally, they had received a great shock. They all described the horror of the dense volumes of smoke, the intense heat and—this was the worst—the groping about in pitch darkness, the electric light being all cut off. The men were troubled a good deal that night by the effects of the foul air on their throats, a horribly scarified feeling, though there was no serious injury. Being anxious to join their families they left the hospital the next day, but they seemed to think it would be a very long time before they got over the horror of the heat and the darkness. The fourth is still in the hospital. He is an old man with both hands badly burnt ; he bears the pain bravely, but he also speaks strongly of the terrible feeling of being shut in with the smoke and the darkness ; for, as the tunnel ends in the station, which is a terminus, and the wind was blowing in from the other end, there was no proper outlet for the fumes which stifled and bewildered the poor sufferers.

### THE RELIGIOUS QUESTION AT LIMERICK.

At a meeting of the Limerick Guardians the question whether a Protestant nurse should be engaged to attend Protestant patients in the workhouse hospital was rather warmly discussed. It was stated that an attempt to obtain a Protestant nurse by means of an advertisement had been unsuccessful, and it was ultimately decided to make a second effort. But is it really necessary that the Protestant patients in the Limerick Workhouse should have a

Protestant nurse? One of the guardians observed that the patients in some of the Dublin hospitals did not know whether they were attended by a Roman Catholic or by a Protestant, and we have no doubt that he is right. There are nurses of all shades of religious opinion in the great London hospitals, but the patients to whom they minister could not easily identify the Church or denomination to which they belong. Of course, there is a sharper dividing line in Ireland, but we do not believe in the necessity for advertising for either Protestant or Roman Catholic nurses.

### UNDERSTAFFING AT COLCHESTER.

The Colchester Board of Guardians are very angry with Captain Hervey, the Local Government Board Inspector, who has reported unfavourably as to the management of the workhouse. But so far as Captain Hervey's remarks upon the imperative necessity of affording assistance to the nursing staff are concerned, no attempt was made at the meeting reported in the local papers to dispute the accuracy of his contention. Indeed, it appears to us that in this respect the inspector errs on the side of requiring too little. He says, in his reply to the strictures of the guardians, that if the average of 15 beds were insisted upon, Colchester would have to increase its nursing staff to nine. But he limits his demand to the appointment of "two good wardmaids," who could relieve the nurses from some of the minor duties they are compelled under present circumstances to discharge. We conclude he has ascertained that this concession would prevent the superintendent nurse from fulfilling her threat to resign on account of the insufficient help afforded her. But it is only a temporary solution of the difficulty, and, as it does not seem to have propitiated the guardians, the inspector himself must be dubious whether his reasonableness was not a mistake.

### THE UNIFORM QUESTION AT EXETER.

Scarcely a couple of years ago the Exeter Board of Guardians passed a resolution to the effect that the uniforms worn by the nurses shall in no case become their property. This decision was arrived at in consequence of conflicting opinions as to the actual ownership of the uniforms. Thus, a nurse who only stayed a few months took her uniform away with her to another situation, and her example was followed by several others in 1890. The changes in the staff being frequent, the charges for uniform became a serious matter, and in order to remove all doubts upon the subject the Guardians adopted the resolution in question. We are surprised that they ever allowed any of the nurses to take away uniforms which, apart from a special understanding, are the property of the Board. But we should have been more astonished if, as a lady member of the Board proposed last week, they had rescinded the resolution. Only herself, the seconder, and one other guardian voted in favour of cancelling it.

### A USELESS REFERENCE.

The Irish Local Government Board seem at last to have convinced some of the Armagh Board of Guardians of the futility of kicking against the pricks. In discussing a sealed order from the Board directing the appointment of a qualified assistant nurse and two wardmaids to the workhouse infirmary, one of the guardians urged that it was useless to act upon a proposition that "the order should be referred to the nursing committee," as there was no doubt that it would have to be carried out. His advice was not followed, but other speakers showed signs of realising the fact that they will be compelled to submit to the inevitable. It is a pity that they have not decided to do so with good grace, instead of trying to maintain a hopeless conflict.

### DISTRICT WORK IN MANCHESTER.

There is but one cause for regret in respect to the annual report of the Manchester and Salford Sick Poor and Private Nursing Institution, namely, that it does not meet with sufficient financial assistance. The record of work done is admirable. The district nursing staff now number 55, who during the past twelve months attended 8,655 patients in their own homes, and paid 222,685 visits. The profit derived from the private nursing department goes towards supporting the district nurses; and the testimony of all sorts of public men in Manchester and Salford shows that the services rendered are of incalculable good. Yet demands for additional help have to be ignored, and the operations of the institution cannot be made to cover the length and breadth of the city owing to the lack of means. The financial resources of the institution have, in fact, been already overtaxed in the effort to maintain the work at its present level. Manchester, however, is a city of such enterprise as well as of such wealth that we cannot believe the appeal for augmented funds on behalf of such an excellent object will continue to be made in vain.

### REDUCING THE STAFF AT PLYMOUTH.

At the last meeting of the Plymouth Board of Guardians the Hospital Committee announced the resignation of two nurses and recommended that one should be advertised for. This raised a storm of opposition, and one of the guardians reminded the board that they decided they would not dismiss nurses, but would not fill vacancies caused by resignations. Notwithstanding the statement that the superintendent nurse had declared that an extra nurse was urgently wanted, in consequence of the number of sick children in the hospital, at least for the winter months, it was decided to make no addition to the present staff of 15 nurses. It is true that this exceeds the requirements of the Local Government Board of one to each 15 patients, but the excess is not necessarily a disadvantage, especially at Plymouth, where if temporary nurses are required they cost 1½ guineas a week as well as board and lodgings. We agree with the contention of the Clerk that, while the Local Government Board stipulate there shall be one nurse to "at least 15 inmates," the guardians should be guided by the advice of the medical officer, who is understood to agree with the superintendent nurse.

### SHORT ITEMS.

Miss Florence Frost, the new matron of the Poor Law Hospital, Salterhebble, Halifax, writes to us to correct the statement that she was in charge of the Riviera Nursing Institute at Nice. She held an appointment on the staff for some time, but the lady superintendent in charge then and now is Miss Godfrey.

### Lectures on Gynæcology for Nurses.

BY ROBERT JARDINE, M.D., M.R.C.S., F.F.P. and S.G., F.R.S.E., Senior Physician to the Glasgow Maternity Hospital, Examiner in Midwifery to the University of Glasgow.

### INTRODUCTORY.

IT is not infrequently said that gynæcological nursing is a somewhat narrow branch of the art of caring for the sick, but with this I cannot agree. A properly-trained gynæcological nurse must have a thorough knowledge of surgical work, especially of that connected with the abdomen. But gynæcological work is not by any means entirely surgical, and she must also be trained in medical nursing. Again, nervous or neurasthenic manifestations are very frequent in cases of chronic pelvic disease, so the nurse must be prepared to take charge of such complications. She will probably find them the most troublesome of all. As a very large percentage of the diseases of the pelvic organs follows abortions, premature, or full-time labours, the nurse, if she is thoroughly to understand her cases, should have a knowledge of obstetric work.

The tendency of the present day is to specialise in all the arts, sciences, and trades, but if a specialist is to be a success, he or she must look beyond the narrow bounds of one particular province. If you wish to be a success, you must guard against narrowing your view to the pelvic organs alone.

As a nurse's duties are exceedingly arduous she must have a good constitution, and she must not forget that her own health is to be considered. At times the strain upon her will be very great, so she must have regular relaxation and rest or she will break down. If she is not in the enjoyment of good health she will not be fit for her duties. A cheerful, sympathetic disposition must be cultivated towards her patients, but in some cases, such as hysterical ones, the sympathy must be tempered with exceeding firmness. A nurse must be free from any manifestation of "nerves." If she cannot control herself under even the most trying circumstances, she has certainly mistaken her vocation. Women are often accused of talking too much; whether or not this is true generally, it is not for me to say, but it cannot be too strongly insisted on that a nurse must not talk too much to her patients. A lady once bitterly complained to me that a nurse who was attending her was constantly starting discussions on Browning. Browning happened to be a favourite author, but the lady did not enjoy discussing him while on a sick bed. To many people in the best of health a discussion of Browning would not have a very tonic effect, and certainly on a sick bed the effect might be disastrous. So-called "shop" talk must be carefully avoided. It is very tempting to relate some of the scenes you have witnessed in hospital; some people like to have their souls harrowed with ghastly tales, but the effect upon an invalid is bound to be bad.

However much a nurse may be tried she should never lose her temper with her patients. Great allowance must be made for people who are ill.

The nurse's duty towards the doctor is plain, viz., to obey all his orders implicitly. Under no circumstances should she criticise his methods of treatment or compare them with those of another. Nurses are occasionally accused of doing this. Such a proceeding cannot be too strongly condemned. The nurse may have been accustomed to very different methods, but she should remember that there are usually more ways than one of treating an ailment and that often very different methods are adopted by different men. She must carry out her instructions to the letter and if bad results follow her conscience will be clear. If she fails to do what she is told or is foolish enough to substitute methods of her own she cannot expect anything else than blame if failure results. While carrying out her instructions she must ever be on the watch for unfavourable indications. On the occurrence of such, she must lose no time in notifying the doctor, but she must be careful not to alarm her patient.

A nurse must be strictly cleanly in her person and dress. The style of dress worn is so well known that we need not discuss it. Bodily cleanliness is very essential, but the hands must be specially clean. Great care must be taken of the nails. They must be kept short and perfectly clean. If the edges round the nails become ragged they must be carefully clipped. A nurse must be very careful if any cracks or fissures form, as she may very easily get blood poisoning. Besides keeping the hands clean, they must also be made comfortably warm before the patient is touched Some people are apt to have cold hands, and if a nurse should be afflicted in this way she must be specially careful not to touch her patient until she has made sure that her hands are warm. This may seem a small matter, but it is well worth bearing in mind. I have had complaints from patients on this point, and in one case the patient suffered so much that she said she would never employ the nurse again, nor could she recommend her to anyone else. In the same way all utensils, such as bedpans, etc., must be warmed before the patient is allowed to use them. It must be borne in mind that when a patient is ill she is likely to be specially sensitive, and instead of adding to her discomfort a nurse must try in every way to sooth her.

If the hands are much in antiseptic solutions they are apt to become chapped, especially in winter weather. A little glycerine and rosewater or lanoline should be rubbed over them, especially at bedtime. It goes without saying that if a nurse gets a poisoned finger she must at once give up attending a surgical case, no matter how slight the septic condition of the finger may be. In the same way if she should have a sore, such as a boil, on any part of her body, she must leave off work. The presence of a small boil, say, on the back of the neck may seem a very trifling thing to put a nurse off duty for, but I can assure you this is not so. A boil is exceedingly septic, and however careful a nurse may be she will be sure to touch it with her fingers. This may be done unconsciously, and virulent septic organisms thus conveyed to the patient.

In conclusion I would like to say a word on menstruation. In the olden days a menstruating woman was considered to be unclean. Among many savage races this idea still prevails. In some tribes the menstruating women are kept apart by themselves, or they have to wear a badge indicating their condition. In the Old Testament strict rules are laid down for the guidance of a menstruating woman, and she is considered unclean during the flow. To the present day Jewish women keep strictly apart from their husbands until they have had the bath of purification seven days after the flow has ceased.

In the present enlightened age we have got rid of many of the absurd notions about menstruation. For instance, we know that the presence of a menstruating woman in a room will not turn wine sour. Yet when we find a belief almost universally held by primitive people it does not do to condemn it off-hand, however absurd it may seem to us. We frequently find that there is a modicum of truth in it.

The practical question is whether a menstruating nurse is likely to be a source of danger at, say, an abdominal section? Some surgeons object to their presence in an operating theatre, and for my part I agree with this. I say that a menstruating nurse should never assist at an operation. In an ordinary sick room there is no danger, provided the nurse is careful. She should wear aseptic diapers, and change them frequently. In doing so she must exercise the utmost care not to contaminate her hands, which she should wash and sterilise just as when dealing with the diapers of her patient.

### Christmas in the Hospitals.

By Our Own Commissioners.

If, in consequence of the prevalence of small-pox, the number of visitors to the hospitals in London was, in the aggregate, less than usual, the efforts of the medical and nursing staff to provide for the entertainment of the patients were never more energetic, nor more successful, than during the Christmas season of 1901. In our issue of December 14th we were able to show how the festival is observed in hospitals beyond the seas. To-day we tell again the old story of how it is kept in the splendid institutions at home which are the parents of those in Greater Britain. It would have been sad indeed if, while the outside interest taken in reconciling the sick and suffering to their detention in the wards at a time when all the world is making holiday has been increasing in the Colonies, it had shown signs of diminishing here. But, happily, the indications are still in the other direction. From the King and Queen and the Prince and Princess of Wales downward, all classes and conditions have manifested a hearty desire to afford practical assistance in the task of contributing to the enjoyment of the inmates. Gifts of luxuries and comforts, of clothing and of toys have never been more numerous, friends of both sexes have never been more ready to proffer their services for the purpose of rendering the Christmas festivities as varied and as effective as possible. In the preliminary and often arduous task of decorating, medical officers and students afforded willing help to the sisters and nurses, who were bent upon transforming the appearance of the wards into that of fairy bowers, and in carrying out the pretty devices which gained so much well-merited admiration. Even in hospitals where elaborate decorations were not attempted, holly and flowers placed on the tables were a pleasing feature. The electric light played an important part, and mitigated apprehensions which on past occasions have in some instances prevailed respecting the perils of using paper decorations. Vastly as the patients appreciate the efforts of the entertainers for their benefit, and look forward to them with keenest delight, their pleasure is enhanced by the care previously exercised in satisfying their material wants; and the Christmas fare, partaken of with scrupulous adhesion to a judiciously-framed scale of diet, paves the way to the contentment of mind, and the elation of spirit, with which they follow the performance of the programme. The diversity of entertainment has been well maintained, and whether the *pièce de résistance* has been the representation of a popular play, the drolleries of a minstrel troupe, or the charming songs of a nurses' glee party, the result has been equally satisfactory. Not only have the hearts of children been gladdened by the provision of Christmas trees of all sorts and sizes, but adults have had their share in the distribution of articles on the heavily laden branches. It has sometimes been suggested that the preparations for the Christmas festivities impose too heavy a burden upon those who necessarily bear the brunt of them. But this view is not endorsed by the willing workers. It is not too much to say that from the matron to the probationer, from the medical superintendent to the rawest of students, there is no one who does not cheerfully contribute to the promotion of the general joy. And, so long as such an admirable feeling prevails inside the hospital walls, the public, to whom the charities look for the means of support and development, may, we think, be relied upon to do their part, no matter how its dimensions extend, in co-operating with them for the purpose of achieving an ideal which is happily not only attainable but is often attained.

### St. Bartholomew's.

Anyone venturing inside the courtyard of Bart's on Christmas Day in the evening would have known that something unusual was going on; the lamps of carriages flashed out into the darkness; figures in caps and aprons with capes over their shoulders flitted across the square, lights shone from every window, and the sounds of singing came from various wards. The work of entertaining was so vast that it was undertaken in groups, each house-surgeon, with his assistants, making himself responsible for his own wards. In one a group of students were singing plantation songs to the accompaniment of banjos, and their programme included a clever sketch of how not to tell lies if you kept a "Nineteenth Century Store." The unhappy property chicken, with his elongated neck, and history of outgrown strength, provoked much mirth; and hearty applause followed the performers as they made their way to the next ward. Harvey Ward had an entertainment by professional niggers, who danced wonderfully, and made their time-worn puns, and the men and boys in this ward thoroughly enjoyed the evening. When the niggers had departed, amid hearty claps, the bran-tub was carried round by two of the nurses, and patients were exhorted to "dip deep down and feel about." "Daddy" being told there were pipes, of course obeyed to the letter, and got his reward in time. A smart red candlestick came to one young fellow who had "no 'ome, worse luck," and it was at once destined for the married sister near King's Cross. "They are good to you in this 'orspital," he added. Altogether, the patients at Bart's had a really good time, and sisters and nurses were well rewarded for the time they had spent tying up parcels and decorating the wards. The coloured lamps were very effective, and there were a few evergreens on the walls. "Ready for bed?" said one of the sisters, about six o'clock. But the answer came, "Not a bit of it!" and decidedly both entertainers and entertained seemed prepared to go on for some time yet. Three grand entertainments will be given in the second week in the New Year; one will be for those patients for whose safe transit doctors and dressers like to be responsible, and the other for the staff. The latter will be of a musical and dramatic character.

### Guy's.

The decorations at Guy's were very graceful and artistic; trailing ivy, fairy lamps, and Chinese lanterns were arranged across the ceilings of the wards, and the convalescent patients, in scarlet jackets, grouped round blazing fires, made a pretty picture. Everyone had a present on Christmas morning, and dinner consisted of roast beef, vegetables, and plum pudding. After dinner came pipes for the men, and in the afternoon a tea was given in the out-patients' department to a large number of poor children, who had their presents from a huge tree. There was a party in Martha Ward on Saturday afternoon, when the nurses brought down their little patients; the children of the men employed in the hospital were also invited and a few friends of the staff. The guests were arranged—in bed, on wheeled carriages or crutches, the children on a square of carpet on the floor, or on the empty beds—all facing the Christmas tree in the corner of the ward, which was lighted with electric light and festooned with glistening glass balls and gay flags. Music preceded a recitation by one of the nurses, and then the whisper, "Here he comes," went round, and in came Santa Claus, hale and hearty, fresh from a snowstorm. "Hands up, all the good boys and girls!" he cried, and of course all the hands went up. The distribution of the presents followed, amid much fun and laughter, until every child had as many toys as he could hold, and everybody had something, if it was only the little brown bear, solemnly presented to the Chaplain of the hospital, or a skipping-rope for one of the students. Sister, nurses, and students were very busy distributing the gifts, and when a piano solo was struck up, it was accompanied by tattoos on dozens of drums and tambourines, till it might almost have been forgotten that this was a ward in a hospital, but for the bandaged heads and pale faces. After the Tree, tea was carried round on little trays, and all who were able feasted on cake and buns. At five o'clock, in the adjoining "Lazarus" Ward, an excellent concert was given, and again the patients were brought in, but this time it was the older ones, men and women who were specially catered for. After a piano solo by Mr. Seymour Dicker, Mr. C. L. Philips gave a violin solo, "Vox Cavatina"; and Sir Liney Levey followed with a comic selection. The great feature of this performance was a song, apparently composed for the occasion, about five little naughty boys who meddled with a motor-car which ran away with them, and took them into Guy's Hospital. Doctors and nurses were brought upon the scene, and there was no more Christmas fare for those unhappy little boys. Mr. J. A. Borrett, whose beautiful singing of "Mary" gained an encore, gave "The Bonnie Banks of Loch Lomond" and an "Evening Song." Miss Gertrude Hunter and Miss Hilda Jackson were the lady soloists, and Mr. Wellbeloved told a terrible story about a horse that was harnessed to a motor-car, but who lost nothing but his tail, while he broke the record for speed. A concert was given in Cornelius Ward at six o'clock, and during the week every ward had a performance of a similar character.

### St. Thomas's.

Santa Claus was in evidence at St. Thomas's Hospital during the early hours of Christmas morning, and the children were the happy recipients of dolls of all sizes and toys of every variety. The wards were decorated with the taste habitually manifested by the nursing staff, Chinese lanterns, ferns, exotics, and evergreen being deftly manipulated to the best advantage. The dinner of the patients on Christmas Day was roast beef and plum pudding, ale and tobacco being also provided. It was not considered necessary to depart from the custom of allowing each patient whose condition permitted to invite a friend to tea. This is a privilege which is immensely appreciated. The usual entertainment by the medical officers and their friends took place in the evening, the round of the wards being perambulated, to the great satisfaction of the patients, who fully entered into the spirit of the music and singing. A Punch and Judy show was a welcome feature on Friday.

### Charing Cross.

Although the patients at Charing Cross Hospital could not have their friends to see them on Christmas Day, they had far from a dull time. Perhaps on this account matron, sisters, and nurses exerted their efforts to the utmost to make the day pass pleasantly. There were more decorations in the wards than was the case last year, and quite a realistic wintry effect was achieved in one or two, with evergreens and cotton wool, even a hot-water coil being transformed into a snowy pillar surmounted by a snowy frog sitting on a snowy toadstool. "So unnatural!" said an ultra-critical patient, which was, of course, an unanswerable objection. The paper flowers, bright lamp shades, and tables loaded with a variety of good things for tea, and the patients themselves sitting up—those who were well enough—in scarlet bed jackets, waited on by doctors and nurses, made a very pretty picture; and the boys of St. Martin's gave great pleasure by singing carols in each ward during the afternoon. Long churchwarden pipes, with permission to smoke, added to the men's enjoyment. Tea was earlier than usual, 'because the friends could not come this year;" this was an "artful dodge" for making the afternoon seem to pass more quickly, and it was evidently very much appreciated. After tea a short entertainment was given in each ward. The wind blew in gusts outside, sending one whole window-sill-full of laurel and art muslin flying; but inside the wards all was bright and warm and Christmas-like, and a patient who was going home next day, but had managed not to miss a Christmas in hospital, owned to having had quite a "gay and giddy time." The Christmas Tree was on Friday afternoon at half-past four. "Christmas makes hard work for the nurses," said the matron, "but it is wonderful what can be done by many hands."

### St. Mary's.

The Christmastide festivities at St. Mary's Hospital, Paddington, occupied the whole of Christmas week to New Year's Day, and during this time each ward had its piano, of which the expense was born by the entertainment fund. The wards were decorated with evergreens by sisters and nurses, assisted by the resident medical officers and students. The Christmas fare consisted of turkeys and plum pudding and cake at the four o'clock tea; but of course many patients were too ill for such sumptuous diet. In the afternoon and evening of Christmas Day the nurses, medical officers, and other friends entertained the patients, of whom there were 271, and each received a present of clothing and a Christmas letter. All the men whose health allowed were given one ounce of tobacco and a pipe, with permission to smoke on Christmas Day and the day after. On New Year's Eve an entertainment for the children took place in the board room. There was first a Punch and Judy show, which always finds great favour with the children; and then in came, not only Santa Claus himself, all scarlet and white, but his two "children," a quaint couple, like a boy and girl from some old picture of half a century ago—the boy with a furious red wig, and the girl with two long flaxen plaits down her back and a strikingly mannish voice. These engaging children helped to distribute the presents, and when at length the last toy was cut down from the tree, and the last cracker pulled, everyone joined in singing "God Save the King." Next Santa Claus called for three cheers for matron and sisters, and was cheered himself, and the fun was over. Then there were oranges for the patients as they walked or were carried from the room, clasping their toys to their hearts. On New Year's Day an entertainment was given to patients (of whom from 100 to 120 were well enough to be present) and the servants of the hospital: this was followed, on January 2nd, by an entertainment for the nurses and their friends. Among those who sent presents for Christmas were the King, the Prince of Wales (President of the hospital), and the Princess of Wales, who sent a case of toys on behalf of her children and the children of Kew. The expenses of all the entertainments were borne by a fund, raised for the purpose, among the board of manage-ment and other supporters of the hospital.

### St. George's.

The Princess of Wales sent a most acceptable gift of clothing to St. George's Hospital, and the lady visitors, in addition, provided presents, and *Truth* and other friends sent toys for the children. On Christmas Day the wards were decorated with evergreens and flowers, and lighted with fairy lamps, and the patients—at least all who were able—had a Christmas dinner of roast beef and plum pudding. On Friday evening a Christmas tea was given in each ward, when every patient received a present, and the children had their Christmas trees, lighted with electric light. On Monday, 30th ult., an entertainment was given in the board room for all who were well enough to go down. The number of patients spending Christmas in the hospital was 290.

THE LONDON.

The Princess of Wales sent a large number of toys for distribution among the patients at the London Hospital, and Prince Edward of York furnished a large toy-boat, which was placed in a prominent position in one of the children's wards. The festivities commenced at six o'clock on Christmas Day morning with the singing of carols in the wards by sisters, nurses, and probationers, some of the patients who were well enough joining in. After breakfast the wards were perambulated by a very different personage, namely, Father Christmas, who freely distributed toys and articles of a useful character. Both turkeys and roast beef figured in the dinner menu. The resources of the hospital are not, however, taxed for the luxuries at Christmas, which are either contributed by friends or paid for out of the ward fund provided by the nursing staff and the medical students. The decorations were practically limited to the adornment of the ward tables with holly and flowers. The feature of Christmas Day was the entertainment given after dinner by the doctors and students, patients too ill to bear the noise being removed to a part of the premises where it did not penetrate. The male patients were permitted to smoke.

UNIVERSITY COLLEGE.

The festivities at University College Hospital commenced on Christmas Eve with a concert in one of the wards in honour of one of the sisters, Miss Riach, who has been appointed by the Colonial Office to undertake nursing duties in one of the Concentration Camps at Pretoria. The whole of the wards were charmingly decorated by the matron, the sisters, and the nurses, and the children were liberally supplied with toys, thanks to the formation of a special fund for the purpose. As usual, there was an abundance of seasonable fare, and in this respect the patients were admirably cared for. The Christmas dinner included turkey and plum pudding, bonbons, fruit, and sweets being afterwards distributed. An interesting feature on Christmas Day was the appearance of a score of members of the choir of a Hampstead church, who sang hymns and carols in the afternoon; while during the evening there was a concert in one of the large wards. On Boxing Day the children in the diphtheria ward had a Christmas tree all to themselves, and on Friday the remainder of the children enjoyed a similar treat. The grown-ups also had their treat on Thursday in the form of a capital concert given by a number of ladies and gentlemen who visited each of the wards in turn.

WESTMINSTER.

The great feature at the Westminster was the variety of bran tubs; each ward seemed to outvie the others in invention. St. Matthew was especially fortunate in being lent the large figure of a peasant woman with a basket on her back, by Messrs. Shoolbred, and in the basket were packed many things to keep the wonder of the patients alive all through Christmas Day, until the time for dips arrived. One very pretty tub was like a rose; indeed, roses blossomed from many unexpected places—from electric lights over patients' beds, from Chinese lanterns hanging from the ceiling, and from the heads of patients and students who had pulled crackers and found strange caps within. Father Christmas visited the wards early in the day, with two stalwart men to help him with his heavy sack; and it was drawing near to the hour for the Christmas Tree when one of the doctors asked a little girl—in bed, and surrounded by toys—what she wanted now, and she truly answered, "Nothing." The men patients perhaps looked further ahead than this small invalid; and when the Matron asked if they had enjoyed their Christmas dinner, the reply was: "We should like the same again to-morrow." For this great feature of the day, one of the students, correctly habited as cook, acted carver; and after dinner smoking began. Then at three o'clock, in Hollond Ward, a beautiful tree was shorn of its fruit; and the children were carried down, including the adored baby, whose eyes gleamed with pleasure when its nurse tied a white woolly hood over its golden curls. Another small but very solemn mite sat clasping an armful of treasures, while he feebly investigated the climbing capacities of his monkey on a stick, and grasped a flag in his thin fingers. After the tree came the entertainment in the board room, for convalescent patients, given by the resident medical staff and friends. The nurses had a busy but a very happy day; and numerous invitations to tea in various wards were given and accepted during the afternoon.

THE CITY OF LONDON LYING-IN HOSPITAL.

In the early morning "sounds of sweet airs" rang through the precincts of the City of London Lying-in Hospital. The sleeping mothers and babes were gently roused by the strain of "Christians, Awake," for the pupils had risen early in order to give the day a good start, and sang lustily several hymns and carols on the staircase. Every mother received a parcel containing a bright red flannel petticoat, a Christmas letter, and last, but surely not least, a complete set of clothing for the babe. Liberty from all the rules was the order of the day. Every mother had chicken, white sauce, and vegetables and plum pudding for dinner. One admitted at 11.30 and confined at 12.15 was busily occupied in picking the wing bone of a chicken at 12.30, and not satisfied with that license, actually ate two helpings of plum pudding and was none the worse afterwards; while the baby, who spent his first Christmas Day in an incubator, is also in first-rate condition. The pupils had an excellent dinner, and showed their appreciation of the good things by giving a hearty three cheers for the matron just outside her door.

BROMPTON CONSUMPTION HOSPITAL.

At the Hospital for Consumption, Brompton, the patients had their usual Christmas fare of turkeys and plum pudding, 20 turkeys being the number required to go round. On Friday evening the Christmas Tree was revealed from behind the curtains of the entertainment room, a vision of glistening fruit; and one feature this year was a useful present of clothing for each patient. Then, of course, came the immortal and wicked "Punch," who pursued his questionable course with his customary vigour, to the accompaniment of laughter and applause. Punch's doctor, who said he was "doing very well, but had no patients," brought the house down; and the riddles which Punch persistently "gave up" were received with shouts of laughter. About half-past seven, the nurses conducted their patients to bed, and the festivities were over for the evening.

NORTH LONDON HOSPITAL FOR CONSUMPTION.

The hospital on Mount Vernon evolved the original idea of having not only a Father but a Mother Christmas, the lady being, as matron explained, the old gentleman's wife, and on Christmas Day these personages distributed gifts to the patients, and toys to be sent to the children who could not be allowed to come this year. After the singing of carols, by matron and nurses at 7 o'clock, there was a celebration of Holy Communion for the patients at 8.30, followed by one for the staff, and a service at 10.30 for all who liked to go. During dinner there was music by friends of the hospital, and the dining-room was gaily decorated with flags. At tea-time came the personages alluded to above, with their gifts, to make their royal procession through the wards, and the patients, who had to be guarded from undue excitement retired early to bed. On Friday evening an entertainment was given, consisting of a concert and an exhibition of shadow pictures.

(*To be continued.*)

### Queen Victoria's Jubilee Institute for Nurses.

Her Majesty the Queen has been graciously pleased to approve the appointment of the following to be "Queen's Nurses," to date January 1, 1902:—

### ENGLAND.

| Nurse. | District Training at | Working at |
|---|---|---|
| Maud Fleming (Supt.) | Manchester | Manchester (Bradford Home) |
| Annie F. Emerson | Kensington | London |
| Josepha Shortt | " | " |
| Annie Boden | Hammersmith | " |
| Amy Lumby | Camberwell | " |
| Lydia B. Thorpe | Haggerston | " |
| Amy Dixon | " | " |
| Edith Ketteridge | Rotherhithe | " |
| Sarah C. Hackett | Paddington | " |
| Annie E. Phillips | " | " |
| Kate E. Bumpus | " | " |
| Eleanor F. Matthews | " | " |
| Annie Mary Sandle | Central Home, London | Barrow-in-Furness |
| Elsie Adelaide Whetham | Bath | Bath |
| Helen S. McDonald | Cardiff | Bilston |
| Ellen Nichols | Brighton | Birkenhead |
| Emilie A. S. Parry | Birmingham | Birmingham (Newhall Street) |
| Margaret E. Williamson | Blackburn | Blackburn |
| Ada E. Morris | Bolton | Bolton |
| Gertrude Webster | " | " |
| Emma Newman | " | " |
| Sarah Annie Pitman | Brighton | Brighton |
| Mary Hitch | " | " |
| Helen Pridham | " | " |
| Edith Ann Gill | Westminster | Bury |
| Sarah A. Thorpe | Central Home London | Cheltenham |
| Sarah C. Parkhouse | Coventry | Coventry |
| Mary G. Hayne | Crayford | Crayford |
| Annie Gledhill | Cardiff | Darington |
| Lucy Blanche Denby | Paddington | East Finchley |
| Ellen St. Clair Woolley | Portsmouth | Gateshead |
| Annie Middleton | Southampton | Gloucester |
| Minnie Snow | Walworth | " |
| Mary E. Fuller | Central Home London | Headington |
| Ethel Annie Lyon | Camberwell | Heywood |
| Louisa E. Young | Liverpool | Horwich |
| Annie Ardagh Thompson | Hammersmith | Hoylake |
| Jessie McRitchie | Hull | Hull |
| Edith Madeleine Wright | Haggerston | Kingston-on-Thames |
| Fanny J. Raikes | Leeds | Leeds (Lovell Street) |
| Millicent Urquhart | " | " " |
| Charlotte E. Tellett | Liverpool | Liverpool (Overton St.) |
| Jessie Robinson | " | " " |
| Sarah Howe | " | " " |
| Hannah M. Parker | " | " " |
| Mary Emma Shaw | " | Liverpool (Central Home |
| Emily Frances Levis | " | Liverpool (Shaw Street) |
| Catherine E. Roberts | " | " " |
| Edith M. Bridges | " | " " |
| Minnie Clark Hamilton | Central Home, London | Longtown |
| Emily Harrison | Manchester | Manchester (Bradford Home) |
| Dora H. Jones | " | " " |
| Edith F. Parker Wake | " | " " |
| May Gale Ware | " | Manchester (Harpurhey Home) |
| Helena Wilson | " | " " |
| Jessie Clara Cunningham | Manchester | Manchester (Hulme Home) |
| Fanny E. Maxwell | Central Home, London | March |
| Josephine Bourdillon | Haggerston | Newmarket |
| Annie Grace Heyward | Sunderland | Norton |
| Lily Elizabeth Hall | Central Home, London | Penshurst |
| Mary Davies | Portsmouth | Portsmouth |
| Julia Gillies | Chelsea | Potter's Bar |
| Mary Dunn | Salford | Radcliffe |
| Jessie Kennett | Chelsea | " |
| Edith Hilda Daniel | Bermondsey | Ramsbury |
| Elizabeth Ellison | Windsor | Rawtenstall |
| Mabel E. Cooper | Portsmouth | Rochdale |
| Clara E. Gore Little | Liverpool | " |
| Marguerite Dancey | East London | St. Helen's |
| Sarah Mills | Salford | Salford |
| Mary Robertson Napier | " | " |
| Martha J. Jones | Haggerston | Shrewsbury |
| Florence J. Sharpe | Hammersmith | Southwell |
| Harriet Hanley | Gloucester | Spalding |
| Lottie Foster | Haggerston | Stafford |
| Elizabeth L. Rose | Sunderland | Sunderland |
| Isobel Hogg Russell | " | " |
| Margaret Cochrane | Torquay | Torquay |
| Lilian Mary Fox | " | " |
| Ellen Rose Wadham | Central Home, London | Truro |

### ENGLAND—*continued.*

| Nurse. | District Training at | Working at |
|---|---|---|
| Millie Eaton | Warrington | Warrington |
| Hannah S. Llewellyn | Cardiff | Warwick |
| Alice Smith | Windsor | Windsor |
| Lilla Young Benson | Worthing | Worthing |
| Elizabeth Lucy Humphrey | Haggerston | Wribbenhall |

### WALES.

| Nurse. | District Training at | Working at |
|---|---|---|
| Violet Ethel Hunt | Cardiff | Cardiff |
| Margaret T. Pritchard | " | " |
| Ellen Lewis | Liverpool | Llangefni |
| Anne Ellen Jones | Camberwell | Menai Bridge |
| Mary Jane Roberts | Salford | Morriston |
| Alice Friend | Walworth | Roch |

### SCOTLAND.

| Nurse. | District Training at | Working at |
|---|---|---|
| Isabella T. Grieg | Edinburgh | Alyth |
| Jessie McDougall Kellie | " | Carluke |
| Martha Ferguson McKee | " | Dalkeith |
| Margaret Ballantine | Dundee | Dundee |
| Mary Inch Steven | Glasgow | Glasgow |
| Euphemia W. Anderson | Edinburgh | Edinburgh |
| Annie Fraser | " | " |
| Annabella Macpherson | " | " |
| Elizabeth Haddon | " | Loch Awe |
| Mary Ann Scott | " | Lochwinnoch |
| Thomasine J. Eustice | " | Port Glasgow |

### IRELAND.

| Nurse. | District Training at | Working at |
|---|---|---|
| Annie E. Irvine | Dublin (St. Patrick's) | Banbridge |
| Elizabeth Johnstone | Dublin (St. Lawrence's) | Dublin |
| Margaret P. Kelly | " | " |
| Mary Rowland Nicholls | Dublin (St. Patrick's) | " |
| Elizabeth M. McKillen | Dublin (St. Lawrence's) | Kenmare |
| Elizabeth O'Shea | " | Kilcommon-Erris |
| Sophie Lampe | Dublin (St. Patrick's) | Newry |
| Caroline Cassidy | " | Portrush |

### Weddings in Our Ranks.

An interesting marriage took place on the 21st ultimo at St. Mary's, Bryanston Square, the bride being Miss L. Margaret Rolfe and the bridegroom Dr. John Middleton Martin. Miss Rolfe was trained at Addenbrooke's Hospital, Cambridge—where she first met Dr. Martin—and at the London Hospital. After taking the course of midwifery at Queen Charlotte's she was appointed sister, and remained for two years in that position. Since then she has held other responsible posts, including that of matron of the Women's Hospital at Brighton. Dr. Martin is Medical Officer of Health for Stroud. After the wedding a reception was held at the Great Central Hotel, where numerous friends assembled, among whom were some former co-workers of both bride and bridegroom well known in their respective professions.

On New Year's Day Miss Henrietta Grace Tillott, of the Army Nursing Service Reserve, was married at Kingsbury-cum-Neasden Parish Church to Mr. Philip John Reid, son of Sir Hugh Gilzean Reid, of Dollis Hill.

### To Nurses.

We invite contributions from any of our readers, and shall be glad to pay for "Notes on News from the Nursing World," or for articles describing nursing experiences, or dealing with any nursing question from an original point of view. The minimum payment for contributions is 5s., but we welcome interesting contributions of a column, or a page, in length. It may be added that notices of appointments, entertainments, presentations, and deaths are not paid for, but that we are always glad to receive them. All rejected manuscripts are returned in due course, and all payments for manuscripts used are made as early as possible after the beginning of each quarter.

### Our Christmas Distribution.

THE following letters have been received by us from the recipients of parcels of garments which we have been able, through the kindness of our readers, to distribute :—

The Matron of the London Hospital, Whitechapel, writes :—"It is with much pleasure that I write to thank you and the readers of THE HOSPITAL for so kindly sending us a Christmas gift of clothes and toys. I can assure you that the clothes will be found most useful, and it will be very nice for our little patients to have the toys. We wish to make it a bright and happy time for our patients at Christmas, and we are grateful to our kind friends for helping us in this."

The Matron of the Metropolitan Hospital, Kingsland Road, writes :—"Many thanks to you and to the readers of THE HOSPITAL for your kind gift of garments for the patients; we are so grateful to you for them, they are all so useful and will be most acceptable to our sick folk; all the garments are so comfortable and well-made and warm, showing that those who sent them knew the requirements of patients in hospital. We appreciate your kind remembrance of us very much, and are very grateful to you."

The Lady Superintendent of the Middlesex Hospital, W., writes :—"I beg to thank the editor and readers of THE HOSPITAL very much for the parcel of very useful things kindly sent for the patients in this hospital."

The Matron of the Royal Free Hospital writes :—"Many warm thanks for a most welcome present for the patients from the editor and readers of THE HOSPITAL."

The Matron of the North London or University College Hospital, Gower Street, writes :—"I beg to acknowledge with very grateful thanks the parcel of warm and useful clothing kindly sent to the patients by the editor and readers of THE HOSPITAL, which will be most acceptable."

The Matron of the Westminster Hospital writes :—"I beg to thank the editor and readers of THE HOSPITAL for their kind gift of clothing, which will be much appreciated by the patients."

The Matron of the Poplar Hospital for Accidents, East India Road, writes :—"Will you please accept our very grateful thanks for the parcel of 13 articles of new clothing which you have so kindly sent for the use of our patients. All the garments will be most useful, but the two flannel shirts that are made to button up the sleeves and on the shoulders will be particularly acceptable in many accident and surgical cases."

The Matron of The Grosvenor Hospital for Women and Children, Westminster, writes :—"I beg to thank you very much for the kind gift of clothing to this hopital."

The Lady Superintendent of the Evelina Hospital for Sick Children, Southwark, writes :—"I beg to acknowledge with grateful thanks a parcel of clothing and a box of bricks for the little sick ones from the editor and readers of THE HOSPITAL."

The Matron of Tottenham Hospital writes : — "Many thanks for the kind gift from the editor and readers of THE HOSPITAL of useful articles of clothing for the patients in the wards. They will be most useful."

The Matron of the North London Consumption Hospital, Hampstead, writes :—"I wish to thank you for your kind gift of clothing, it will be very much appreciated."

The Matron of the North-Eastern Hospital for Children, Shoreditch, writes :—"The little patients thank the editor and readers of THE HOSPITAL for the nice Christmas gifts which they like very much."

### Appointments.

CHILDREN'S HOSPITAL, MIDDLESBROUGH. — Miss A. B. Todd has been appointed matron. She was trained at the Birmingham Infirmary, and was afterwards for four years on the nursing staff of St. Bartholomew's Hospital, London.

FOYLE HILL INFECTIOUS DISEASES HOSPITAL, LONDONDERRY.—Miss Fanny Sheldon has been appointed matron nurse. She was trained at the Liverpool Workhouse Infirmary, and has since been engaged as district nurse under the auspices of the Queen Victoria Jubilee Institute.

FULHAM INFIRMARY.—Miss Elizabeth Howlett has been appointed superintendent of night nurses. She was trained at Southwark Infirmary, East Dulwich, for three years, and has since been ward sister first at Aston Infirmary, and subsequently at Bethnal Green Infirmary.

MILDMAY INSTITUTE, NEWINGTON GREEN. — Miss Ellemine B. E. Gjellestad has been appointed nurse on the private staff. She was trained for three years at Poplar and Stepney Sick Asylum, Bromley, E., has been nurse at Bergen General Hospital, Norway, and assistant nurse at the Northern Fever Hospital, Winchmore Hill.

ROYAL BOSCOMBE AND WEST HANTS HOSPITAL.—Miss Helen Hortin has been appointed staff nurse. She was trained at the Hospital for Sick Children, Great Ormond Street, London, and the Royal Victoria Hospital, Bournemouth. She has been on the private staff of the Warneford Hospital, and assistant nurse in that institution.

ROYAL NAVAL HOSPITAL, HASLAR.—Miss Rosa Northcote has been appointed nursing sister. She was trained for three years at the Brentford Union Infirmary, Isleworth, and has since been nurse, and also for a few weeks staff nurse, at the Miller Hospital, Greenwich. Miss Northcote holds the certificate of the Incorporated Society of Trained Masseuses.

ROYAL UNITED HOSPITAL, BATH.—Miss L. Marian Wales has been appointed matron's assistant and Miss Florence B. Mathews night sister. Miss Wales was trained at the Bristol General Hospital. She was subsequently on the private staff of the Bristol General Hospital for two years, sister in charge of theatre and the female wards for one year and ten months at the Dorset County Hospital, and night sister at Royal United Hospital for nearly two years. Miss Mathews was trained at St. Bartholomew's Hospital.

ROYAL VICTORIA HOSPITAL, BELFAST. — Miss Mary Florence Bostock was appointed matron. She was trained at Leeds General Infirmary, and has since been head nurse at Barbados General Hospital, in charge of wards at Leeds General Infirmary and Cardiff Infirmary, and lady superintendent at Throne Hospital, Belfast.

SPITTLESEA INFECTIOUS DISEASES HOSPITAL.—Miss Susan Jacob has been appointed matron. She was trained at Sir Patrick Dun's Hospital, Dublin, and has since been matron at the County of Meath Infirmary, assistant matron at Wolverhampton Borough Hospital, and matron at Lowestoft Sanatorium.

WOODSTOCK HOSPITAL, NEAR CAPE TOWN.—Miss Evelyn Hawes has been appointed matron. She was trained at Mill Road Infirmary, Liverpool, and has since held the appointment of matron at the Victoria Hospital, Lovedale, South Africa, for two and a half years.

### Wants and Workers.

WOULD any nurse like for poor patient a bamboo bedrest and walnut bed-table, both in serviceable condition, though not new? They would be sent carriage paid. Address Miss A. E. Briggs, 9 St. Stephen's Square, Bayswater, W.

WOULD anyone kindly send THE HOSPITAL on the Monday after publication on payment of three months' postage in advance? Address District Nurse, South Brent, Devon.

### Echoes from the Outside World.

THE departure of the King and Queen on Monday for Sandringham is a fact gladly chronicled. Although the reports of the Queen's illness stated that she was only suffering from a chill, chills have so often grown rapidly into something worse that it is hardly a matter for wonder that a measure of uneasiness was felt by the public. This was, perhaps, really due to the knowledge that the Queen only reluctantly decided to be away from her Norfolk home on Christmas Day. However, she was in time to celebrate the entrance of the New Year by a family gathering, and with her accustomed kindness spent some of her time while in confinement at Marlborough House in dictating a message to the Limehouse waifs hoping that they might enjoy the hospitable liberality provided for them by the "Children's Sunbeam Society" of South Australia. No wonder the children applauded lustily when they heard the message, for they had been warmed with fire, fed with beef and plum pudding, and anon cheered not only by the fact that 11,000 little colonials were caring for them, but that their Queen, too, was thinking of her ragged little subjects.

UNTIL the morning of New Year's Day arrived and no list of honours was published, it was hardly realised that a long-established custom had been abolished. Not that the present Sovereign is likely to be more chary in awarding justly earned distinctions than any of his predecessors. There has merely been an alteration in dates. In the reign of Queen Victoria the 1st of January and her birthday in May were the recognised times for distributing honours in the bulk. During the reign of King Edward his birthday in November and Coronation Day will be substituted. Of course, there is a little disappointment this week amongst the people who expected, perhaps without any reason, to figure in the New Year's List.

EVIDENTLY English ideas and American notions differ on the exact meaning of the word "small." This is the adjective applied on the invitation cards to the dance given to mark the *début* of Miss Alice Roosevelt. The number of guests invited to the White House for January 3rd is thirteen hundred, which one would have imagined would have been sufficient to constitute a big ball. The festivity is given, not officially by the President of the United States, but by Mrs. Roosevelt on her daughter's behalf, and the President's name does not appear on the cards at all. The gathering is likely to be one of the most brilliant social functions of late years, and Miss Roosevelt will be able to enjoy the proud distinction that she is the first girl who has made her *début* at the historic mansion. By the way, Mrs. Roosevelt and some other leaders of society are endeavouring, in the interests of hygiene, to introduce a new article of dress. It is a dinner jacket for keeping the shoulders warm when sitting in a room where the temperature is low. It is to be made of beautiful silken material, and to be modelled upon the one worn by the wife of Charles II. in the celebrated painting.

I HAD occasion two or three days after the accident at Liverpool to travel by the South London electric line, and I could not help reflecting as I looked round at my fellow passengers, most of whom were reading quietly, or chatting with friends, how short a time it takes for a disaster to be forgotten. As far as can be ascertained, the fire on the Liverpool Overhead Railway did not cause one single passenger the less to take tickets on the electric railways in London. A few may have reflected that at no time is the chance of a catastrophe so remote as when one has just occurred, but probably most of the passengers gave no thought to the possible failure of the current. If a calamity of this sort should occur on either of the "Tubes," it is devoutly to be hoped that it may not be between 8 and 10 A.M., or 5 and 7 P.M., since owing to the shameful overcrowding the loss of life would be fearful. On the ordinary railways it is exceedingly difficult to prevent extra passengers "squeezing in," because it is impossible for the guard to visit each carriage at each station; but there is no excuse on the electric lines, where a man is in charge of every compartment, and could easily close the gates when the full complement of passengers had entered if he were bidden to do so. There is one lesson that all passengers can learn from the evidence given at the Board of Trade inquiry at Liverpool. The signalman dipped his handkerchief in water, and put it into his mouth, and thus, he believes, saved his life; and a lad named Gough, only 16 years of age, was able to go into the dense smoke and guide several passengers to the platform who would otherwise have perished in the smoke, because previous to entering the tunnel he had stood under a water tap and drenched himself.

THE children's banquet at the Guildhall on Tuesday, when upwards of a thousand poor and crippled little ones from all parts of London dined in the Guildhall, was preceded at one o'clock the same day by a dispatch of hampers to many thousands who, owing to infirmity, could not be present. My invitation card was as follows:—"Ragged School Union and Shaftesbury Society.—The company of ——— is desired at the dispatch of Sir William Treloar's hampers to 5,000 London cripple children by the Right Hon. the Lord Mayor, from Guildhall Yard." The Lord Mayor, not being able to greet these children in person, chose the carmen of 20 vans to represent him, as it were, in deputy by conveying boxes of materials for a banquet in their own homes to his absent guests. Each parcel contained a meat pie, a cake, a Christmas pudding, tea, and sweets, given, too, with no grudging hand, but sufficient in quantity to feed many, if not all the little brothers and sisters of the tiny host or hostess. The banquet in the evening was a remarkable success. During the proceedings a message was despatched to the King and Queen wishing them "A Happy New Year," who telegraphed a gracious acknowledgment.

LAST year at this time Ping Pong was almost unknown; this year not only has the familiar box containing net, bats, and balls been given as a Christmas present to thousands of glad receivers, but the popularity of the game has been proved by the fact that a tournament, in which there were a large number of entries, was concluded at Queen's Hall on Saturday afternoon. The prizes were of a most substantial character, that won by the first lady being a splendid silver tea and coffee set, value £25. There was an enormous concourse of people to witness the semi-final and final rounds, and the combatants could not complain of any lack of enthusiasm, for the applause which greeted a "telling" or a clever stroke was loud and stimulating. A smart little boy, Master Muir Stephens, about thirteen years old, carried off the third prize, and once astonished everyone by an over-the-shoulder stroke, and a good deal of the play of the elders was uncommonly neat and even elegant. As far as could be judged by Saturday's and the previous contests, a decided preference is being shown by latter-day players for the wooden rather than the vellum-covered bat. It is not so pretty a weapon, much resembling the back of a hair-brush, but it is found to "drive" better, and the more easily ensures a quick return. They, however, neither "ping" nor "pong." The gentlemen played in their shirt-sleeves, but the ladies, by special request, wore dark blouses. It was found that whereas the white of the gentlemen's sleeves in no way interfered with the aim of the opponent, the brighter hues of the ladies' light blouses was detrimental to the vision, and made the white ball difficult to see. This little fact is worth remembering by those who are likely to take part in a Ping Pong Tournament—and nurses are great players. Unless you wish to feel that you are cordially disliked by your prospective opponent, don't wear a blouse of brilliant hue.

### Everybody's Opinion.

[Correspondence on all subjects is invited, but we cannot in any way be responsible for the opinions expressed by our correspondents. No communication can be entertained if the name and address of the correspondent are not given as a guarantee of good faith, but not necessarily for publication. All correspondents should write on one side of the paper only.]

### THE "ENGAGED" PROBATIONER.

"BEBE" writes: I cannot agree with the doctor who would refuse to allow an engaged girl to enter nursing ranks. When taking up nursing, we have, to a certain extent, to give up dancing, theatres, and parties, for they are apt to interfere with our professional work. But what difference can an engagement make? Letters can be written, or meetings arranged during the periods of off-duty. Is it natural for any girl to have no interest outside her hospital? Surely, she would be quickly "down in the dumps," and if a nurse does her duty well and conscientiously, does it matter to others that she is engaged? And should not the future wives know how to nurse their children? How many children do we come across who suffer from ill-health through ignorance on the part of the mother. In my opinion, all girls should have a practical knowledge of nursing, therefore, let each have as good a training as she can get, since a little knowledge may prove a dangerous thing.

### NURSES AND THEIR FOOD.

"A FORMER SISTER" writes from Pau: I am quite in sympathy with those night nurses who complain of being neglected as regards variety in food. I have done a great deal of night duty during ten years' experience as a nurse. At one hospital we had an egg every night to take on duty with us for the three months we were on duty, and at subsequent places it has always been the same, with sometimes cold meat as an alternative. It reminds me of the old woman who said:—

For Sunday's dinner I can boast
I have a leg of mutton, roast,
On Monday if the truth be told,
I eat it with some pickles cold,
On Tuesday, I some slices fry,
On Wednesday, I may make a pie.
On Thursday, I, to cut a dash,
Do make of it a savoury hash.
And that my meat may longer last
On Friday I proclaim a fast.
On Saturday it has got so narrow,
I crack the bones and eat the marrow.

I think that far too many matrons of hospitals emulate this old woman's example.

### THE SCARCITY OF WORKHOUSE NURSES, AND THEIR SUPPOSED TROUBLES UNDER THE MASTERS AND MATRONS OF WORKHOUSES.

"A TRAINED WORKHOUSE MATRON" writes: I have watched with much interest the letters which have from time to time appeared on the above subject, and think some of them libellous in the extreme. To be a workhouse matron is synonymous to being a vulgar, ignorant, and illiterate woman in the eyes of a large section of the nursing world. Some there may be, but it is not true of them as a class Are there not some nurses whose education is not all that could be desired? Hitherto masters and matrons have been very apathetic about the reports which their anonymous traducers have circulated, but I think that the one by "Another Workhouse Nurse" in your issue of December 14th should not be allowed to pass unnoticed. I should like to know how many masters and matrons she has lived with! Could she substantiate her statements *re* the unkindness of the masters to the inmates? How much personal experience has she had of the inner workings of the house and infirmary, and what are the particular things which go on behind the scenes? It seems to me clearly the duty of "Workhouse Nurse," if she is cognisant of such flagrant abuse of their duties and responsibilities on the part of masters, to make it known to the Local Government Board, and have such unsuitable ones weeded out. Why does not she and other correspondents behave like Britons, if they are such; show that innate love of truth and fairplay which we are said to be endowed with; state the particular unions, giving dates and circumstances, instead of writing letters of such a vague, defamatory nature as some of them have been? No one would then doubt their honesty of purpose in propagating such statements. Unless accounts of a number of L.G.B. enquiries are soon published, masters and matrons will conclude that the letters are written with a malicious intention to damage their reputation as a class, and that the statements have no foundation in fact. Some correspondents have been charitable enough to confess that a trained nurse, if she were matron, would behave differently to the staff; but a trained matron is not always able to satisfy the class of women who occupy the position of nurse in some workhouses. I know of several trained matrons of workhouses whose experience of nurses since they left their training schools and had anything to do with small workhouses leads them to think that the character of a large number of nurses has been faithfully described by "Yorkshire," "Lancashire," and "Matron;" as soon as some women put on cap and apron they set to work to see how many causes of complaint they can discover. There is a growing tendency on the part of nurses to criticise adversely all those who have any authority over them, whether they are their professional superiors or not. "Workhouse Nurse" advises a Poor-Law Guardian to look at both sides of the question. Has she done so herself? I should not think so! In all controverted questions it behoves us to understand clearly the positions of either party, and to weigh calmly the statements advanced. Evidently, reading between the lines of all the letters, the correspondents wish to make it clear that they think the matron belongs to "the lower order" (a term frequently made use of by nurses), and that they (the nurses) belong to the well-bred, well-educated class. I should imagine some of the writers are of the type of woman the matron of the Metropolitan Hospital had in mind when she said "it was easier to get a good nurse than a good woman." Ah! how true one feels that to be as experience of nurses increases. I know a trained matron must understand the requirements of the infirmary better than an untrained one. Still, I am convinced that the causes mainly responsible for the dearth of nurses in small workhouses are, viz., the old, rambling, badly-ventilated buildings, ill adapted for modern nursing, poor apartments, inadequate salaries, lack of acute cases of illness, and, what an inspector has lately pointed out, indifference on the part of some doctors to the needs of the sick, consequently causing the nurses to lose interest and the work to become more monotonous than it should be. Some of these causes are to be found in every workhouse. I could enumerate more, all of which have nothing to do with the nurses relating to the master and matron, but it would only be waste of time. If the supply were equal to the demand, and all these causes were removed, even though the untrained matron remained, I feel sure we should hear less about "The Scarcity of Workhouse Nurses."

### Presentations.

FORD INFIRMARY, DEVONPORT.—At the close of a concert held in the infirmary, Devonport, on December 23rd, Miss Nicholls, the superintendent, was presented by the doctor and nursing staff with a handsome cut-glass and silver scent-bottle, and a bottle of perfume.

THE nurses of the Trained Nurses' Institute at Weymouth have given the superintendent a cut-glass smelling bottle with silver top, and the matron a silver table gong, with which they were very pleased.

### For Reading to the Sick.

THE NEW YEAR.

*As thy days thy strength shall be.*

O LORD, fulfil Thy Will
Be the days few or many, good or ill:
Prolong them, to suffice
For offering up ourselves Thy sacrifice;
Shorthen them, if Thou wilt,
To make in righteousness an end of guilt.
Yea, they will not be long
To souls who learn to sing a patient song;
Yea, short they will not be
To souls on tiptoe to flee home to Thee.
O Lord, fulfil Thy Will:
Make Thy Word ours, and keep us patient still,
Be the days few or many, good or ill.

*Christina G. Rosetti. Verses.*

---

If we see not what lies beyond, let us walk on where we see and we shall reach it.—*E. B. Pusey.*

---

Do not look forward to the changes and chances of this life in fear; rather look to them with full hope that, as they arise, God, whose you are, will deliver you out of them. He has kept you hitherto—do you but hold fast to His dear hand, and He will lead you safely through all things; and, when you cannot stand, He will bear you in His arms. Do not look forward to what may happen to-morrow; the same everlasting Father who cares for you to-day, will take care of you to-morrow, and every day. Either He will shield you from suffering, or He will give you unfailing strength to bear it. Be at peace then, and put aside all anxious thoughts and imaginations.—*Francis de Sales.*

---

We have been placed upon the Way. We have been taught the Truth. We have been made partakers of the Life. The Way must be traversed; the Truth must be pursued; the Life must be realised. Then cometh the end. Our pilgrimage, long as it may be or short, if we have walked in Christ will leave us by the throne of God; our partial knowledge, if we have looked upon all things in Christ, will be lost in open sight; our little lives, perfected, purified, harmonised in Him Whom we have trusted, will become in due order parts of the one Divine Life when God is all in all.—*Bishop Westcott, The Revelation of the Father.*

---

We tell Thee of our care,
Of the sore burden pressing day by day:
And in the light and pity of Thy face
The burden melts away.

We breathe our secret wish,
The importunate longing which no man may see;
We ask it humbly, or, more restful still,
We leave it all to Thee.

The thorns are turned to flowers;
All dark perplexities seem light and fair;
A mist is lifted from the heavy hours,
And Thou art everywhere.

*Susan Coolidge.*

### Notes and Queries.

**The Editor is always willing to answer in this column, without any fee, all reasonable questions, as soon as possible.**
**But the following rules must be carefully observed:—**
**1. Every communication must be accompanied by the name and address of the writer.**
**2. The question must always bear upon nursing, directly or indirectly.**
**If an answer is required by letter a fee of half-a-crown must be enclosed with the note containing the inquiry.**

---

*Ear-cap.*

(127) Some 50 years ago a friend of mine had an ear-cap which he placed behind his ear to assist him in hearing; is such an ear-cap in use now? If so, where can one be procured?—*E. R.*

Any surgical instrument maker would give you the information.

*Spinal Curvature.*

(128) Some time ago I remember seeing in one number of THE HOSPITAL an account of an apparatus designed for the relief of spinal curvature. I have not the back numbers of the paper, and would be much obliged if you would kindly tell me in which it appeared.—*E. C. T.*

You will find what you want in THE HOSPITAL of October 5.

*Orphanage.*

(129) I am a widow and have struggled hard to bring up my boys well, even at the sacrifice of my resources. I realise that one between eight and nine years would be better in stronger hands, and I am, therefore, anxious to get him placed in an orphanage where he would be taught a trade. He is a promising boy, and I should feel much indebted to you if you could give me a list of suitable institutions.—*M. H.*

A list of the principal orphanages is given in "Burdett's Hospitals and Charities." You might apply to the secretary of the British Orphan Asylum, 62 Bishopsgate Street Within, E.C., and to the secretary of the London Orphan Asylum, 21 Great St. Helens, Bishopsgate Street, E.C.

*Mentally Unhinged.*

(130) I want to find a temporary home for a poor seamstress who is overwrought, and, therefore, mentally unhinged. The case is urgent, as if the patient does not recover, though she is perfectly harmless, she would have to be sent to an asylum.—*J. H.*

There is need of homes where persons mentally unhinged could be kept awhile, under supervision, until either the malady declared itself, or the patient recovered. The secretary of the Metropolitan Asylums Board, Victoria Embankment, E.C., or the secretary of the After-care Association for Poor Persons Discharged Recovered from Asylums, Church House, Dean's Yard, S.W., might be able to recommend a home.

*Uniform.*

(131) Can you tell me what uniform I should have to provide on entering a hospital as probationer?—*Ruby.*

Your matron will give you particulars. The uniform varies in different institutions.

*Stipulated Rules.*

(132) 1. Are there any stipulated rules for the management of private nurses' homes? I have one, and keep a staff of six trained nurses for private work. One of them is delicate, and has been unable to take a case for weeks. 2. Am I expected to keep her until she is well again and to pay her medicine bills? It is a great expense.—*Anxious Matron.*

1. No. 2. Yes, unless you have a written agreement to the contrary.

*Anatomy.*

(133) Could you kindly suggest books on human anatomy (not elementary) suitable for a prospective student of medicine?—*Jerza.*

Gray's "Anatomy" is a standard work on the subject, and the new edition is up to date.

---

**Standard Books of Reference.**

"The Nursing Profession: How and Where to Train." 2s. net; post free 2s. 4d.
"Burdett's Official Nursing Directory." 3s. net; post free, 3s. 4d.
"Burdett's Hospitals and Charities." 5s.
"The Nurses' Dictionary of Medical Terms." 2s.
"Burdett's Series of Nursing Text-Books." 1s. each.
"A Handbook for Nurses." (Illustrated). 5s.
"Nursing: Its Theory and Practice." New Edition. 3s. 6d.
"Helps in Sickness and to Health." Fifteenth Thousand. 5s.
"The Physiological Feeding of Infants." 1s.
"The Physiological Nursery Chart." 1s.; post free, 1s. 3d.
"Hospital Expenditure: The Commissariat." 2s. 6d.
All these are published by the SCIENTIFIC PRESS, Ltd., and may be obtained through any bookseller or direct from the publishers, 28 and 29 Southampton Street, London, W.C.

